# Cultural and Contextual Adaptation of an eHealth Intervention for Youth Receiving Services for First-Episode Psychosis: Adaptation Framework and Protocol for Horyzons-Canada Phase 1

**DOI:** 10.2196/resprot.8810

**Published:** 2018-04-23

**Authors:** Shalini Lal, John Gleeson, Ashok Malla, Lysanne Rivard, Ridha Joober, Ranjith Chandrasena, Mario Alvarez-Jimenez

**Affiliations:** ^1^ School of Rehabilitation Faculty of Medicine University of Montreal Montreal, QC Canada; ^2^ Health Innovation and Evaluation Hub University of Montreal Hospital Research Center Montreal, QC Canada; ^3^ Douglas Mental Health University Institute Montreal, QC Canada; ^4^ School of Psychology Australian Catholic University Fitzroy Australia; ^5^ Department of Psychiatry McGill University Montreal, QC Canada; ^6^ Chatham-Kent Health Alliance Chatham-Kent, ON Canada; ^7^ Centre for Youth Mental Health The University of Melbourne Melbourne Australia; ^8^ Orygen, The National Centre of Excellence in Youth Mental Health Parkville Australia

**Keywords:** mental health, young adult, telemedicine, eHealth, social support, therapy, psychology

## Abstract

**Background:**

eHealth interventions have the potential to address challenges related to access, service engagement, and continuity of care in the delivery of mental health services. However, the initial development and evaluation of such interventions can require substantive amounts of financial and human resource investments to bring them to scale. Therefore, it may be warranted to pay greater attention to policy, services, and research with respect to eHealth platforms that have the potential to be adapted for use across settings. Yet, limited attention has been placed on the methods and processes for adapting eHealth interventions to improve their applicability across cultural, geographical, and contextual boundaries.

**Objective:**

In this paper, we describe an adaptation framework and protocol to adapt an eHealth intervention designed to promote recovery and prevent relapses in youth receiving specialized services for first-episode psychosis. The Web-based platform, called Horyzons, was initially developed and tested in Australia and is now being prepared for evaluation in Canada.

**Methods:**

Service users and service providers from 2 specialized early intervention programs for first-episode psychosis located in different provinces will explore a beta-version of the eHealth intervention through focus group discussions and extended personal explorations to identify the need for, and content of contextual and cultural adaptations. An iterative consultation process will then take place with service providers and users to develop and assess platform adaptations in preparation for a pilot study with a live version of the platform.

**Results:**

Data collection was completed in August 2017, and analysis and adaptation are in process. The first results of the study will be submitted for publication in 2018 and will provide preliminary insights into the acceptability of the Web-based platform (eg, perceived use and perceived usefulness) from service provider and service user perspectives. The project will also provide knowledge about the adaptations and process needed to prepare the platform for evaluation in Canada.

**Conclusions:**

This study contributes to an important gap in the literature pertaining to the specific principles, methods, and steps involved in adapting eHealth interventions for implementation and evaluation across a diverse range of cultural, geographical, and health care settings.

## Introduction

### Background

eHealth interventions have the potential to address challenges related to access, service engagement, and continuity of care in the delivery of mental health services [1,2]. However, the initial development and evaluation of such interventions can require substantive amounts of financial and human resource investments to bring them to a level of scale. It is therefore important to consider alternative avenues to advance the practice of eHealth in a global context. One possibility for health system planners and service providers is leveraging the existing eHealth interventions that have been developed and tested in one country or jurisdiction and adapting these interventions for use in different communities and settings. This would reduce duplication of efforts at a global scale in terms of spending public, industry, and philanthropic resources to produce seemingly novel services and products that have already been developed elsewhere. It would also be in alignment with how health care innovations have traditionally been scaled up. However, more attention on how best to transport promising eHealth innovations across geographical, cultural, and contextual boundaries is warranted.

There are two general approaches to transporting eHealth interventions from one context to another: adoption and adaptation [3]. Adoption refers to a direct transport of the intervention by the developer or importation by the new context without systematically or extensively considering how the innovation fits with the needs and characteristics of the local service context, culture, and population. Adaptation involves making changes to the intervention to increase its fit with the local population and setting in which it is to be newly implemented and tested for its efficacy and effectiveness. It involves, for example, consideration of language, culture, and context [4,5]. Building on the definition of intervention adaptation from Sundell et al [3] and cultural adaptation from Bernal et al [4] and Castro et al [5], we define *eHealth adaptation* as the systematic, purposeful, and collaborative process of making changes to increase the relevance and acceptability of an eHealth innovation to a local population and health care setting and ultimately increase its effectiveness.

Thus, the purpose of adapting an intervention is to ensure that it is meaningful and satisfactory for a population that is different from the population for which the intervention was originally developed [6]. It has been suggested that acceptability of an intervention may influence the extent to which individuals engage with and participate in an intervention, which can ultimately affect its effectiveness [5]. Moreover, engaging in an adaptation process could help increase ownership of the intervention by the local setting and improve its sustainability especially if conducted using a collaborative and shared decision-making process [3,7]. Interventions that are matched in terms of linguistic, educational, and developmental needs of a population, and that have content that is perceived as interesting, useful, and relevant to a population’s everyday life may elicit higher levels of engagement, and ultimately contribute to effectiveness [3].

In fact, recent systematic reviews indicate that the effectiveness of mental health–related interventions (in-person or Web-based) developed in one context and evaluated in another, are influenced by whether the intervention was adapted before the implementation [3,6,8,9]. In other words, those studies that involved cultural and contextual adaptations of interventions yielded larger effect sizes when compared with interventions that were not adapted for the same clientele group; however, it is important to note that these results have not been consistent across studies. As such, scholars have argued for more research to determine the effect of adaptations when transferring interventions across populations, settings, and countries [3,5]. This can help in determining whether the benefits of adaptation are worth the costs [5] and also to begin identifying what types of adaptations contribute the most toward outcomes.

Part of the challenge in reviewing the effectiveness of intervention adaptation is that there is limited consensus on the methods and processes for adapting an intervention. This is particularly the case for the eHealth field as many of the adaptation frameworks that have been developed were originally meant for face-to-face interventions. For example, Harper Shehadeh et al [6] highlight that some of the elements proposed in the cultural adaptation framework documented by Bernal and colleagues [4] may not be applicable in minimally guided interventions (including those delivered online). The cultural adaptation framework documented by Bernal and colleagues is widely cited in the psychosocial intervention literature pertaining to cultural adaptation. A second challenge in this field is limited documentation on adaptations undertaken by researchers when transporting or importing innovations from one context to another. For example, 2 recent systematic and meta-analytic reviews [3,6] on the adaptation of mental health interventions, including those delivered online, found few studies reporting details on the methods and processes of adaptation. This type of information is needed to inform the interpretation of findings as well as implementation and scale-up [6]. The lack of documentation on adaptations undertaken may in part be due to the dearth of guidance on the methods, processes, and impact of adapting eHealth interventions when implementing them across populations, settings, and countries.

In summary, more information and research attention are needed on the steps involved in transporting or importing interventions from one context to another, as well as the influence of adaptation on acceptability and outcomes. Toward this end, we report on an eHealth adaptation research framework and protocol for a Web-based intervention developed in Australia, Horyzons, in preparation for a pilot implementation study in Canada.

### Description of the Intervention

Horyzons is a Web-based therapeutic platform designed to sustain the treatment benefits of specialized services for psychosis especially in relation to relapse prevention and social functioning through the provision of psychosocial interventions during transitions from specialized to regular mental health care. Horyzons was originally developed in Australia by coauthors (A2, A7), an interdisciplinary team of experts (professional writers, clinical psychologists, comic developers, artists, experts in computer science and human computer interaction), and codeveloped with young people who have received specialized services for a first-episode psychosis (FEP). The team worked iteratively over a 30-month period following participatory design principles, positive psychology, evidence-based interventions (eg, mindfulness), and strengths-based models [10,11]. The Web-based portal consists of interactive strengths-based psychosocial interventions, peer-to-peer Web-based social networking, as well as clinical and peer moderation to provide guidance and ensure safety. The moderation approach of the intervention is informed by self-determination theory and supportive accountability to enhance engagement with the Web-based intervention and motivation in social and psychological functioning [12,13]. It has been tested on a sample of 20 young Australian adults for its feasibility, acceptability, utility, and safety [10,14]. Further details on the Horyzons platform and its core features are provided in Textbox 1 and illustrated in Multimedia Appendices 1 and 2.

Our aim is to create an adapted version of Horyzons that is tailored for a Canadian young adult population. It could be argued that cultural, language, and contextual differences between Westernized countries such as Australia and Canada are minimal as, for instance, both countries have publicly funded health care and a high proportion of cultural and ethnic diversity. However, there are differences in relation to how mental health services are implemented at the front-line, which may influence implementation of the intervention. Moreover, Canada has a history of both French and British colonization whereas Australian colonial history is British, which highlights the importance of attending to communication practices and related linguistic considerations in the context of transporting or importing an eHealth intervention.

The adaptation of the Horyzons platform is the first step in a multiphased, international research program.

**Overview of the Horyzons system.**

**Purpose:** To promote long-term recovery in youth with psychosis.

**Developers:** Multidisciplinary development team comprising of software developers, mobile developers, novelists, comic artists, clinical psychologists, experts in human computer interaction, experts in machine learning and natural language processing, young people with lived experience.

**Original population:** Youth with first-episode psychosis (FEP) living in Melbourne, Australia.

**Main components:**

- Therapy modules (steps): discrete, interactive, evidence-based therapy modules addressing, for example (1) personal strengths (eg, identifying personal strengths via an interactive card-sort game based on the strengths-based frameworks; (2) mindfulness (eg, activities to enhance self-compassion); and (3) connecting with others (eg, modules providing guidance on how to respond to the good news expressed by others; how to respond empathically to others). Content is conveyed through text, video, audio, and interactive visual graphics.
- Persuasive system features (“do its,” “playlist”) to promote behavioral change: behavioral prompts that support the implementation of a “step” in real-life contexts (eg, following a step about identifying personal strengths, the user is prompted to exercise a core personal strength such as kindness in specific contexts such as at school or work). A “playlist” stores and schedules any “do-it” the young person wants to complete in the future. Behavior change is also promoted through social network features described below.
- Social network features (“the café,” “team up,” “talk it out”): Users are encouraged to communicate with one another through the Web-based social network or “café” to foster social support and connectedness. Each user creates her or his own profile with images (as on Facebook) and can visit the wall of fellow users, where their posts and general activity are displayed. Users can rate, comment on, and share any step with others via the social networking newsfeed. Users can also support others’ efforts to engage in specific behavioral changes via the “team up” function. A group problem-solving function (Talk it out) aims to promote social self-efficacy and interpersonal problem solving. It allows users to nominate issues (eg, “how to deal with low self-esteem about your body?”).
- Moderation (“expert moderators” and “super-users”): Expert moderation is by mental health professionals experienced in treating patients with psychosis. Their role is to provide guidance, monitor participants’ clinical status, and ensure the safety of the social network. Each expert moderator is assigned a caseload (a full-time moderator can manage 100 users). Expert moderators develop brief case formulations that are presented at weekly supervision meetings with senior clinicians. Moderators send each client tailored content suggestions weekly based on the clients’ needs, interests, and strengths. Suggestions appear on the user’s home page, and they receive a text notification via an inbuilt text-messaging (short message service) function. Super-users are young people with lived experience of FEP who have received peer-support training. Their role includes providing support and fostering engagement (eg, reaching out to reticent users, posting “ice-breakers,” commenting and liking posts, and modeling activity). The site is monitored by moderators 7 days a week during moderating hours. The system is set up to send automatic notifications to the moderator when posts have words that could be indicative of risk; these posts are blocked until the moderator can assess the post and its related risk following a clinical safety protocol.

Specifically, the aim of this phase I study is to assess the initial acceptability of the platform by analyzing perspectives of Canadian young adults receiving specialized services for FEP and service providers on the Horyzons platform (eg, in relation to perceived usefulness and ease of use), and to adapt the platform in preparation for evaluation. The results will then inform the design for phase II, a pilot test of the adapted platform with a small sample of mental health service users. Subsequently, these results will support detailed planning for controlled evaluations of the intervention (eg, randomized controlled trial).

### Conceptual Framework

To inform and guide the adaptation, we conducted a literature review on research pertaining to adapting interventions across cultural and contextual settings. We identified several models on the adaptation process developed across a range of different fields (eg, psychology, education); for example, in relation to psychotherapy and evidence-based health interventions [4,15-23]. We also identified frameworks evaluating Web-based tools [24,25], implementation research models (eg, Revised Ottawa Model of Research Use) [26], and technology and innovation models (eg, technology acceptance model) [27]. Some of the key elements drawn from this review are summarized in the sections below followed by a presentation of the eHealth adaptation framework that will be used in this study.

According to Castro et al [5], there are 3 broad elements that need to be considered when adapting interventions: characteristics of the population that will receive the intervention; the staff that would be involved in delivering the intervention; and other administrative, contextual, and community factors. Where there are mismatches between aspects of the current intervention with these 3 elements, adaptations should be considered. Moreover, several models suggest a staged process for adaptation that incorporates qualitative and quantitative data over the course of a series of steps leading to changes in an intervention [5,15,16]. A synthesis of these models suggests the following steps that are important to consider in the adaptation process: (1) assess and generate knowledge from target population, program implementers, and stakeholders; (2) determine the need to adopt or adapt the intervention; (3) identify elements to adapt, respect core elements of the original intervention, pilot-test adaptations with target population, program implementers, and stakeholders; (4) integrate adaptations into the intervention; (5) conduct a formal evaluation of the adapted intervention (eg, pilot study); (6) refine the intervention if necessary; and (7) conduct efficacy, effectiveness, and implementation trials.

In terms of specific elements to assess, the Ecological Validity Model from Bernal et al [4] suggests culturally sensitive elements, such as language, concepts, and content, to address during the adaptation process, whereas the Framework for Evaluating the Quality of Multimedia Learning Resources [24] and the Mobile Assessment Rating Scale [25] identify several items that help evaluate users’ experiences of a Web-based platform, for example, motivation, aesthetics, accessibility, interaction, quality and credibility of information, and usability. The Revised Ottawa Model of Research Use [26] highlights the importance of considering barriers and supports, including the adaptors, the practice environment, and implementation strategies. Finally, the technology acceptance model suggests that the perceived ease of use and perceived usefulness of an information technology will influence users’ acceptance of a technology [27].

Moreover, before engaging in an adaptation of an eHealth intervention, it is important to clarify the therapeutic intervention principles upon which it is based, including what the intervention aims to achieve and how it achieves it [28]. For example, Horyzons is based on supportive accountability principles that highlight that human support (ie, social presence of an individual online that is seen as trustworthy and having expertise, such as a clinician moderator) increases motivation to engage in the intervention, which is important for clinical outcomes [13]. Thus, to maintain internal validity of an eHealth intervention, adaptations would not focus on the core principles (eg, supportive accountability through clinician moderation) but rather on content and features of the platform to enhance its usability, interactivity, relevance to a local population and context, and alignment with current technologies.

These aforementioned principles and models were used to develop our research framework, which is organized into the following 3 objectives and stages: (1) to assess initial perspectives of service users and providers of the eHealth intervention (without any modifications) following a brief overview and interaction with the website, (2) to assess perspectives of the eHealth intervention after an extended exploration, and (3) to adapt the eHealth intervention based on feedback from key stakeholder groups (while respecting its core therapeutic elements and principles, and considering feasibility of adaptations in terms of resources available). The second objective is important as it provides an opportunity for participants to explore the platform in the community at their own pace and more extensively, which could help elicit additional perspectives pertinent for adaptation. Further details on the eHealth adaptation framework in terms of methods and processes are provided in Table 1. All stages of the study seek to better understand participant perspectives of all 4 components of the platform. We anticipate that most of the adaptations that will be recommended will be in relation to the therapeutic modules; however, this remains to be assessed based on the results of the study.

**Table 1:** eHealth adaptation framework. ICT: information and communication technology; N/A: not applicable.

Framework components			Stage 1	Stage 2	Stage 3
Objectives			To assess initial perceptions and experiences of the original Web-based platform from the perspectives of service users and service providers following a brief overview, including the core therapeutic principles and features it is based on, and exposure to it.	To assess perceptions and experiences of the original Web-based platform after engaging with it over a duration of 2 weeks.	To adapt the Web-based platform based on stakeholder feedback, while respecting its core elements and feasibility of resources.
Questions			What are the perceptions of mental health service users and service providers regarding the Web-based platform?	What are the perceptions of mental health service users and service providers regarding the Web-based platform following an extended personal exploration of 2 weeks?	What are the recommendations of mental health service users and service providers on adapting the Web-based platform to enhance its relevance and acceptability?
Topics			Language; culture Likes, dislikes, facilitators, barriers Usefulness Safety Design, ease of use Therapeutic alignment Organizational factors	Language; culture Likes, dislikes, facilitators, barriers Usefulness Safety Design, ease of use Therapeutic alignment Organizational factors	Targeted features and content for adaptation based on stage 1 data.
Participants			Service users, service providers	Service users, service providers	Service users, service providers
**Data collection**					
	Activity		ICT use survey, platform introduction, brief exploration and guided activities, feedback forms, group discussion	Extended exploration and feedback forms	Consultation meetings
	**Methods of collection**				
		Service providers and users	*Survey*: Topics include Access to technology Use of Internet and related technologies Barriers and facilitators to using technology *Focus group*: Questions include What do you like the most about the platform? What do you dislike the most about the platform? How is the content helpful? (probe: images, audio, videos) How are the features helpful? What are your thoughts on: how motivating and engaging it is to use; what could hinder motivation to use the platform; how safe the platform is to use; the support that is offered on this platform; the design and layout; and, how easy it is to use? If you could make one change or add something to the platform, what would it be? Please identify any words, expressions, or parts of the platform that seem unclear What are your suggestions for adaptations (eg, in relation to language, metaphor, images, ease of use, content, the way information is presented)?	N/A	Identify modules, features, content, activity that needs adaptation. For each adaptation, the following topics will be discussed in an iterative manner Relevancy or fit for Canadian context Written feedback on each adaptation (likes, dislikes, content difficult to understand) Content, links to add or delete; general comments
		Service providers only	N/A	Explore 4 features, activities, modules to evaluate (2 preselected by the researchers based on service provider expertise and 2 selected by the service provider). Complete written feedback form for each activity identified in terms of suggested adaptations; reasons why they are important; general comments.	N/A
		Users only	N/A	Explore 4 features, activities, modules to evaluate (2 preselected by the researchers and 2 selected by the service user). Complete written feedback form for each activity identified in terms of content, sentences, or words that are not clear or difficult to understand: What you like?; What you dislike?; and general comments. Additional questions: Which other activities did you try on the website?; What are your comments related to these other activities (eg, likes, dislikes, recommendations for changes)?; Please share any other comments or suggestions.	*Questionnaire*: Would you recommend the use of this platform to others? (5-point Likert scale) Please elaborate Would you feel safe on this platform? Y or N—Please elaborate Please list 3 key obstacles that might prevent an individual from using this platform Please list 3 strategies that would encourage a young person to use this platform What would be an ideal total number of participants using this platform at the same time? How would you use this platform? Please select from the following: Learning about what happened to you, and how to get better; Social networking (meeting other youth that have gone through similar experiences; Support from other youth; Support from mental health service providers; Other purpose, specify

## Methods

### Participants and Setting

This research will take place in 2 specialized early intervention clinics for FEP, 1 urban and 1 semirural, located in different provinces. Both programs provide a comprehensive range of services for young people diagnosed with FEP and follow best practice guidelines for real-world settings [29,30]. Service users and providers from both sites will take part in the study. Eligibility criteria for service users are as follows: diagnosed with a psychotic disorder, within their first 3 years of treatment and currently followed by a clinician, considered to be symptomatically stable and capable of participating in focus groups as judged by their primary treating clinician, and 18 years of age and older. Eligible service providers include psychiatrists, case managers, or other health care professionals with a minimum of 2 years of experience working in the field of specialized early intervention for FEP and regularly involved in delivering services to youth with FEP. We aim to recruit a minimum of 11 service providers (6 urban and 5 rural) and 11 service users (6 urban and 5 rural).

Ethics approval has been obtained from the ethics review board of the primary recruitment site and from the ethics review board of the secondary site. All participants will provide written, informed consent before participating in the study.

The same data collection activities will be conducted at both sites and with both groups separately on a beta-version of the eHealth intervention: sociodemographic and technology use questionnaire, focus group discussions with written feedback forms, and extended explorations of the platform with written feedback forms.

### Stage 1: Assessing Initial Perspectives and Experiences of the eHealth Intervention

To address the first objective, service users and providers will respond to a survey and take part in focus group sessions. In addition to collecting sociodemographic information, the survey will assess participants’ access, experiences, and attitudes on their use of information and communication technology (ICT) as well as their use of ICT in relation to obtaining mental health information, services, and supports. The focus group sessions with service users and providers will take place separately in a computer lab with approximately 4 to 5 participants per session and will last approximately 90 min. Each participant will be assigned to their own computer station (or laptop) and logged into the platform as an individual user with a personalized access code. The focus groups will be led by 2 facilitators and will include a brief tutorial on the core features of the intervention, for instance, the persuasive system, the social networking features, the role of moderators, the main content areas, and the core therapeutic principles. The facilitator will also describe how the platform is envisioned to be implemented in the future pilot study and a subsequent randomized controlled trial. The aim of this first step is 2-fold: first, to help participants understand the intention and functioning of the platform as well as the overall vision for its future implementation before considering local and contextual adaptations; and second, to provide participants with a general understanding of how the website is organized to facilitate their subsequent individual explorations of the website. Next, participants will individually explore the platform at their workstations and provide feedback on the following: general impressions (likes, dislikes, questions, comments); usefulness, safety and support; design, layout, and navigations; ease of use; and suggestions for modifications and adaptations (Canadian context and language). Patient participants will receive $25 CAD, and clinician participants will be offered lunch for their participation in this component of the study.

### Stage 2: Assessing Perspectives and Experiences of the eHealth Intervention After an Extended Exploration

At the end of the focus group session, service providers and service users will be invited to individually explore a beta version of the eHealth intervention for a maximum of 120 min over a 2- to 4-week period from a personal computer. Each participant will be given personal log-in information (username and password) to access the platform providing them with the opportunity to continue their exploration of the platform’s therapeutic content and activities. Participants will be given either an email or electronic copy of a feedback form with detailed instructions and questions to capture their impressions and suggestions for modifications and adaptations. Patient participants will receive $50 CAD for their participation in this component of the study.

Data obtained during stages 1 and 2 of the research will provide insights on whether the platform and its aims as a whole are understandable and whether participants would be interested in using the platform as a complement to the services they receive. The qualitative data from the focus groups will be recorded and transcribed verbatim, and the written feedback responses (from the focus groups and extended explorations) will be organized into tables. The data will be managed using Atlas.ti (Scientific Software Development GmbH, Version 7.5.6), and a coding framework will be developed based on the interview guide and a thematic analysis approach. The quantitative data will be assessed using descriptive statistics. In line with the convergent mixed-methods model, the quantitative and qualitative data will first be analyzed separately and then considered for an integrated analysis of the findings [31].

### Stage 3: Adapting the eHealth Intervention

The adaptations identified in stages 1 and 2 will be considered in relation to the feasibility of making the adaptations as well as how they might affect the core features of the intervention (eg, fidelity). The adaptations that are suggested by participants will be discussed with the intervention authors to assess the extent to which these adaptations would affect fidelity of the platform. Moreover, results from this initial adaptation study will provide insights on strategies that may need to be implemented to ensure fidelity of the intervention during the pilot implementation.

If needed, an additional process of consensus discussion will be added to prioritize the adaptations that will be pursued. Service providers who take part in the focus groups will first be consulted on an individual basis to identify the details of the adaptations that will be made. Service users will be invited to assess the usefulness and accessibility of the adapted content and to share their feedback. Patient participants will receive $25 CAD for their participation in this final stage of the study. The adapted eHealth intervention will be further assessed during the second phase of the research program, that is, a small pilot study using a live version of the site. A live version of the site will provide participants access to the full range of social media features of the platform such as communicating with others, as well as posting images, videos, and links. Moreover, a live version would also provide access to a Web-based peer support worker and clinician moderator.

## Results

The project was funded March 2015 and data collection was completed in August 2017. Analysis and adaptations are currently under way, and the first results are expected to be submitted for publication in 2018.

## Discussion

### Study Rationale and Significance

This protocol addresses an important gap in the eHealth intervention literature in terms of frameworks, methods, and processes used by researchers to adapt an eHealth intervention before its implementation and evaluation in different contexts and settings. Although more research is needed on the effectiveness of adapting interventions, there is a general consensus by several authors of systematic and meta-analytical reviews of psychological and health-promotion interventions (eg, [3,6]) that considering adaptation when transporting interventions and programs is well-warranted. Moreover, not only is there a “moral case to test and demonstrate the appropriateness, acceptability, and harmlessness of interventions up front” [6], there is increasing evidence indicating that such a process can positively impact the effectiveness of an intervention. We believe this would extend similarly to the adaptation of eHealth innovations when considering their implementation across geographical, cultural, and contextual settings, particularly those that are based on psychological and social therapy principles and interventions.

Access to details on the adaptation of eHealth interventions is important for supporting the interpretation of results obtained from effectiveness studies when an innovation is implemented and studied across different settings. Although the importance of retaining therapeutic principles and mechanisms of an intervention when scaling up evaluation are recognized, there are limited examples of how changes to increase relevancy, alignment with current technologies, interactivity, and fit can be considered when scaling up evaluation of eHealth innovation across cultural and contextual settings. The eHealth adaptation framework that we have developed for this study provides a concrete example of the process and methods for how adaptations to psychological, social, and educational interventions in the field of eHealth could be addressed. It highlights the importance of considering the therapeutic principles and mechanisms upon which the intervention is based; the population, the service providers, and the setting (eg, urban, rural, program delivery model) in which the innovation will be implemented; extended opportunities to explore the eHealth intervention at a pace that is more reflective of real-life implementation; considering the perspectives of different stakeholder groups, including their experiences, skills, access, and attitudes toward the use of technology; and, providing different media and methods through which to collect data pertaining to adaptation (eg, focus groups, written feedback, consultation).

In addition, we will be able to compare adaptations suggested across settings (eg, urban, multiethnic vs semirural). Our framework also attends to the various types of media that participants can access on the website, including the visual images, audio, and video supports, and the importance of understanding whether these media are presented in a manner that is relatable to different audience groups. For example, given that the graphics are created by an Australian visual artist, it is possible that some of the imagery and symbolism may not be readily understandable to the Canadian population. Also, the voice recordings for therapeutic activities (eg, mindfulness, breathing) have an Australian accent, and this might hinder participation and engagement. The study will also provide information on words that may need to be added or rephrased in accordance with Canadian English language. The system has a database of synonyms obtained from Web-based dictionaries and can make suggestions on aspects of the website that may be of interest to participants based on their posts (by matching words used in their posts and related synonyms from the dictionary to therapeutic content on the website). As such, it will be important to assess the appropriateness of the dictionary being used by the system during the adaptation of the intervention.

### Limitations and Future Research

Certain limits of the adaptation protocol are also acknowledged. For example, the protocol invites participants to reflect on features of the platform that would be available during the live implementation (eg, clinician moderator, peer support worker); in this regard, some of their perspectives will be based on projections into the future, rather than actual experience. To mitigate this limitation, we plan to pilot-test the adapted version, with all features accessible, with a small sample (n=20) of participants over a period of 8 weeks. The pilot study will also have a qualitative component assessing participants’ experiences of all aspects of the platform, for example, interactions with peers, peer support worker, and clinician moderators. This will facilitate obtaining additional data on the acceptability of the eHealth intervention and any further adaptations needed before conducting controlled evaluations.

### Conclusions

The results of this study will provide preliminary insights into the acceptability of the Horyzons Web-based platform (eg, perceived use and perceived usefulness) and knowledge about the adaptations and process needed to prepare the platform for evaluation in Canada. Moreover, this protocol contributes to an important gap in the literature pertaining to the specific principles, methods, and steps involved in the adaptation process for scaling up evaluation of eHealth innovations. This type of research is novel from a Canadian and international eHealth perspective and is increasingly relevant in a global environment where eHealth innovations are being considered for implementation across a range of cultural, geographical, and health system contexts.

## Appendix

Multimedia Appendix 1 Horyzons' Screenshot: How Horyzons Works.

Multimedia Appendix 2 Horyzons' Screenshot: Strengths.

## Abbreviations

FEP: first-episode psychosis
ICT: Information and communication technology
