# Supplementary figures and images for: Cultural and Contextual Adaptation of an eHealth Intervention for Youth Receiving Services for First-Episode Psychosis: Adaptation Framework and Protocol for Horyzons-Canada Phase 1
Source: JMIR Res Protoc. 2018 Apr 23;7(4):e100. doi: 10.2196/resprot.8810 (PMC5938599; doi:10.2196/resprot.8810)

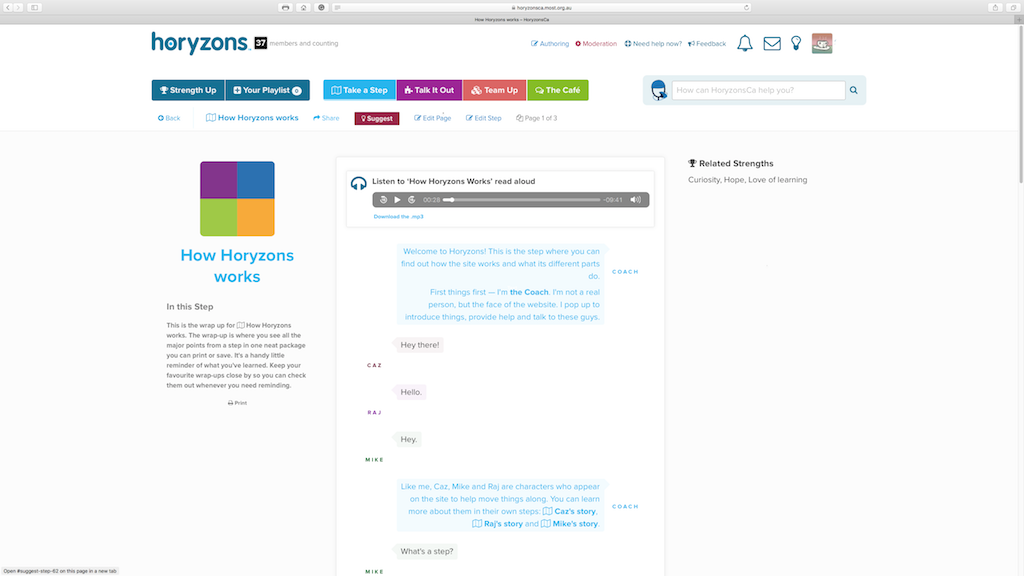

Supplement: Multimedia Appendix 1 [file resprot_v7i4e100_app1.png]

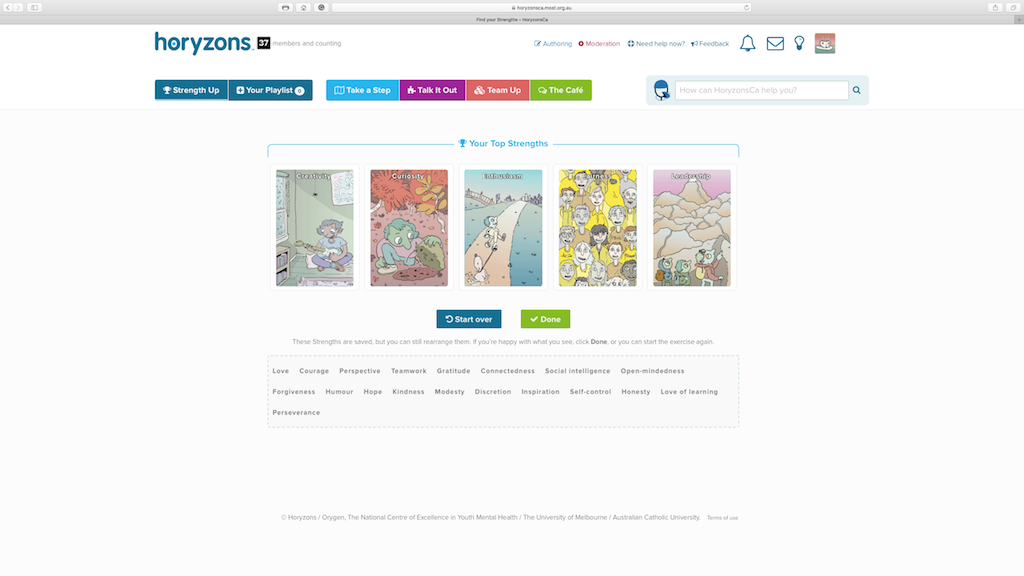

Supplement: Multimedia Appendix 2 [file resprot_v7i4e100_app2.png]
